# Serine protease inhibitor Kazal type 1 (SPINK1) drives proliferation and anoikis resistance in a subset of ovarian cancers

**DOI:** 10.18632/oncotarget.5927

**Published:** 2015-09-30

**Authors:** Christine Mehner, Ann L. Oberg, Kimberly R. Kalli, Aziza Nassar, Alexandra Hockla, Devon Pendlebury, Magdalena A. Cichon, Krista M. Goergen, Matthew J. Maurer, Ellen L. Goode, Gary L. Keeney, Aminah Jatoi, Miklós Sahin-Tóth, John A. Copland, Derek C. Radisky, Evette S. Radisky

**Affiliations:** ^1^ Department of Cancer Biology, Mayo Clinic, Jacksonville, FL, USA; ^2^ Division of Biomedical Statistics and Informatics, Department of Health Sciences Research, Mayo Clinic, Rochester, MN, USA; ^3^ Department of Medical Oncology, Mayo Clinic, Rochester, MN, USA; ^4^ Department of Laboratory Medicine and Pathology, Mayo Clinic, Jacksonville, FL, USA; ^5^ Division of Epidemiology, Department of Health Sciences Research, Mayo Clinic, Rochester, MN, USA; ^6^ Division of Anatomic Pathology, Department of Laboratory Medicine and Pathology, Mayo Clinic, Rochester, MN, USA; ^7^ Department of Molecular and Cell Biology, Boston University Henry M. Goldman School of Dental Medicine, Boston, MA, USA

**Keywords:** SPINK1, ovarian cancer, serine protease inhibitor, EGFR, anoikis

## Abstract

Ovarian cancer represents the most lethal tumor type among malignancies of the female reproductive system. Overall survival rates remain low. In this study, we identify the serine protease inhibitor Kazal type 1 (SPINK1) as a potential therapeutic target for a subset of ovarian cancers. We show that SPINK1 drives ovarian cancer cell proliferation through activation of epidermal growth factor receptor (EGFR) signaling, and that SPINK1 promotes resistance to anoikis through a distinct mechanism involving protease inhibition. In analyses of ovarian tumor specimens from a Mayo Clinic cohort of 490 patients, we further find that SPINK1 immunostaining represents an independent prognostic factor for poor survival, with the strongest association in patients with nonserous histological tumor subtypes (endometrioid, clear cell, and mucinous). This study provides novel insight into the fundamental processes underlying ovarian cancer progression, and also suggests new avenues for development of molecularly targeted therapies.

## INTRODUCTION

Ovarian cancer represents only 5% of all malignancies in women, but has the highest number of cancer related deaths among malignant tumors of the female reproductive system; the 5-year survival rate of 45% has changed little over the past few decades [1]. The majority of patients are diagnosed with advanced disease (stage 3 or 4). These patients undergo cytoreductive surgery and most will initially respond to first-line chemotherapy, typically platinum agents with taxanes, but almost all will have recurrence of chemoresistant disease [2, 3]. The ovarian cancer field has not yet incorporated molecularly targeted therapies into standard treatment, and trials of a few initially promising agents have been disappointing [3, 4]. Efforts to identify new therapeutic targets and to understand the molecular pathways by which they contribute to tumor growth and progression are warranted, along with accompanying methods for predicting patient response and stratifying patients for treatment regimens based on molecular cancer drivers [4].

Serine protease inhibitor Kazal type 1 (SPINK1), also known as pancreatic secretory trypsin inhibitor (PSTI), has been best characterized as an inhibitor of digestive trypsins secreted by the pancreatic acinar cells into the pancreatic juice; by inhibiting intra-pancreatic activation of trypsins SPINK1 prevents autodigestion of the organ [5]. Also designated as tumor-associated trypsin inhibitor (TATI), SPINK1 was initially purified from the urine of a patient with serous ovarian adenocarcinoma [6]; very high concentrations of SPINK1 have also been observed in benign and malignant mucinous ovarian cyst fluids, with lower concentrations reported in cyst fluid from other ovarian tumor types [7]. SPINK1 has subsequently been found to be secreted into the blood and urine by a variety of other tumor types [8]. Recent studies of SPINK1 in different cancers have focused on tumor tissue staining of SPINK1, and on the potential functional effects of SPINK1 on tumor growth and progression. Studies in prostate, colon, pancreatic, and breast cancer models have provided evidence that SPINK1 can promote cancer growth and progression [9-14]. Based on results from these models, it has been proposed that at least some of the protumorigenic functions of SPINK1 are not mediated through its classical activity as a serine protease inhibitor, but rather through alternative mechanisms of action. One potential mechanism implicated in prostate and pancreatic cancers involves direct stimulation of cell signaling pathways through interaction with the epidermal growth factor receptor (EGFR) [10, 11, 13]. These findings have led to suggestions that intervention in SPINK1 signaling may represent a useful therapeutic strategy for subgroups of cancer patients with SPINK1-driven tumors [10, 15].

In ovarian cancer, expression of SPINK1 in tumor tissue was previously found to be associated with poorer survival in a group of 56 stage 3 and 4 ovarian cancer patients [16]. This finding has yet to be validated in other cohorts, and it remains unclear what the prognostic significance of tumor SPINK1 may be across a wider range of ovarian tumor histological types and stages. Importantly, the potential functional role of SPINK1 in ovarian cancer growth, progression, and metastasis has not been explored previously; here we interrogate the function of SPINK1 towards identification of potential points of intervention for molecularly targeted therapeutic strategies in ovarian cancer.

In this study, using cell culture models of invasive epithelial ovarian cancer, we find that SPINK1 directly promotes ovarian cancer cell proliferation and resistance to anoikis. We also evaluate tissue expression of SPINK1 in a patient cohort of nearly 500 women with invasive epithelial ovarian cancer, comprehensively assessing prognostic significance in relation to stage, grade, and histological subtypes. Our results implicate SPINK1 as a driver of proliferation and as a potential therapeutic target in a subset of ovarian cancers spanning multiple histological types.

## RESULTS

### SPINK1 drives proliferation in ovarian cancer cells

**Figure 1:**
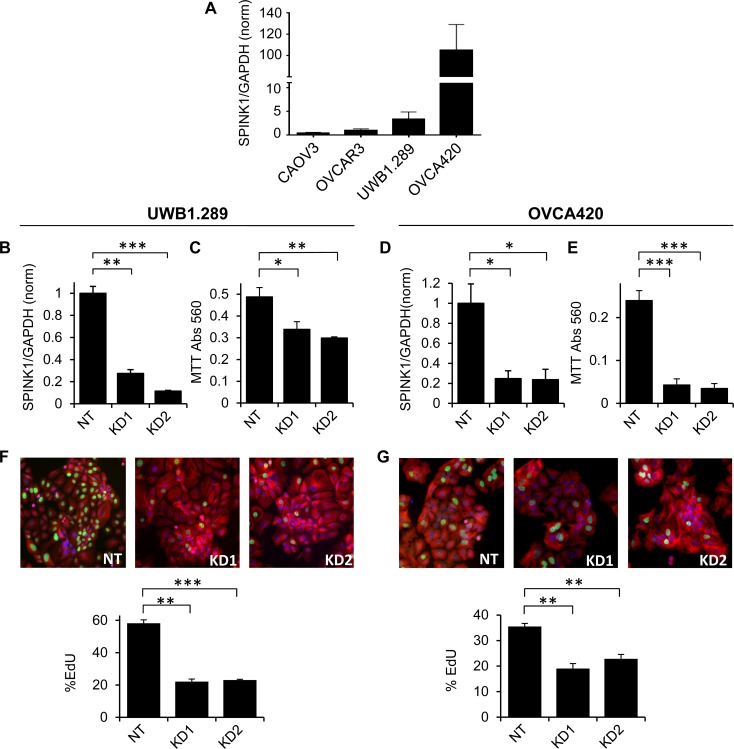
Endogenous SPINK1 expression drives proliferation of ovarian cancer cells **A.** SPINK1 transcripts were measured in ovarian cancer cell lines by qRT/PCR normalizing to GAPDH; values on y-axis show expression relative to OVCAR3. **B.** UWB1.289 cells transduced with shRNA lentiviruses KD1 and KD2 targeting SPINK1 show effective knockdown relative to cells transduced with non-target control virus (NT), as assessed by qRT/PCR (KD1 *p* = 0.0005, KD2 *p* = 0.0001). **C.** SPINK1 knockdown in UWB1.289 cells results in significant reduction in metabolically active cells as assessed by MTT assay (KD1 *p* = 0.0527, KD2 *p* = 0.0115). **D.** OVCA420 cells transduced with shRNA lentiviruses KD1 and KD2 targeting SPINK1 show effective knockdown relative to cells transduced with non-target control virus (NT), as assessed by qRT/PCR (KD1 *p* = 0.0228, KD2 *p* = 0.0258). **E.** SPINK1 knock-down in OVCA420 cells shows significant reduction in metabolically active cells as assessed by MTT assay (KD1 *p* = 0.0001, KD2 *p* = 0.0001). **F.** SPINK1 knockdown in UWB1.289 cells results in significantly reduced proliferation in EdU assay (KD1 *p* = 0.0002, KD2 *p* = 0.0001). **G.** SPINK1 knockdown in OVCA420 cells results in significantly reduced proliferation in EdU assay (KD1 *p* = 0.002, KD2 *p* = 0.0013). *p < 0.05; **p < 0.01; ***p < 0.0001 (unpaired *t*-test).

To assess the potential functional importance of SPINK1 for ovarian cancer cell growth, we initially identified four commonly used epithelial ovarian cancer cell lines spanning a broad range of endogenous expression levels of SPINK1 as assessed by qRT/PCR of transcripts (Figure 1A). We also assessed SPINK1 protein in conditioned medium by enzyme-linked immunosorbent assay (ELISA), measuring 157 pg/ml (25 pM) in conditioned medium from the highest expressing cell line, OVCA420, whereas SPINK1 in lower expressing cell lines did not reach the detection limit of the assay. Using lower and higher expressing cell lines UWB1.289 and OVCA420, respectively, we silenced SPINK1 gene expression using two different shRNA constructs (Figure 1B, 1D) and measured effects on cell growth. For both cell lines, suppression of SPINK1 expression resulted in a significant reduction in cell growth compared to the non-target control as assessed by MTT staining (Figure 1C, 1E).

The MTT assay provides a comparative measure of the number of viable and metabolically active cells, reflecting the balance of cell proliferation and cell death. To measure the specific effect of SPINK1 on ovarian cancer cell proliferation, we next carried out EdU incorporation assays. For both the UWB1.289 and OVCA420 cell lines, we observed significantly reduced EdU incorporation in cells in which SPINK1 expression was suppressed (Figure 1F, 1G) establishing a functional role for endogenous SPINK1 in promoting proliferation of ovarian cancer cells. Intriguingly, both cell lines show strong dependence of proliferation on SPINK1 despite varying expression levels, suggesting that SPINK1 can provide a powerful growth stimulus even when present in low concentration.

**Figure 2:**
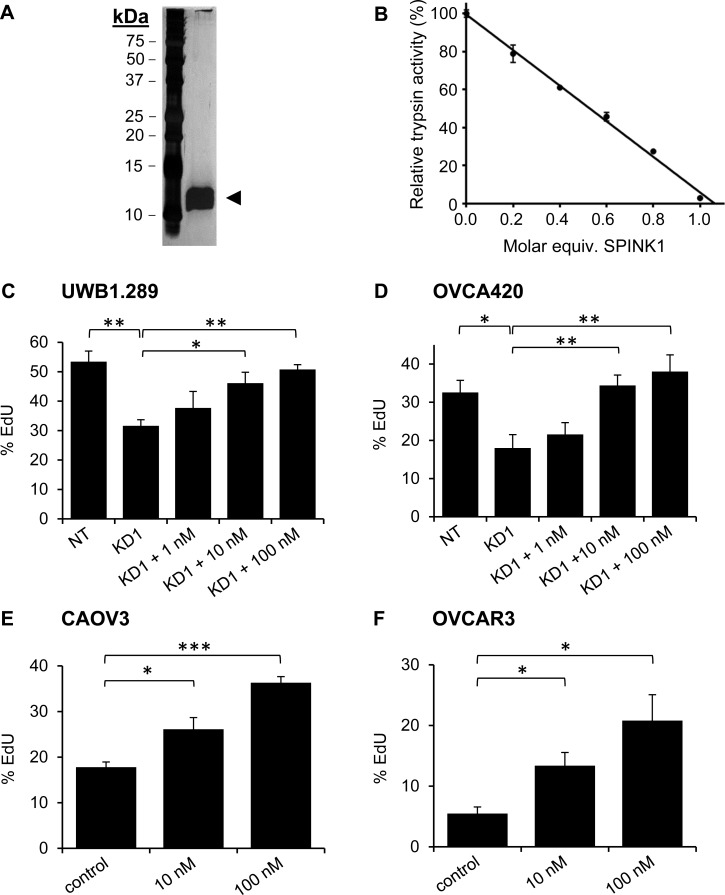
Recombinant SPINK1 stimulates proliferation of ovarian cancer cells **A.** Silver stained gel shows highly purified rSPINK1 protein following expression in HEK293E cells and purification via affinity chromatography and gel filtration. **B.** rSPINK1 shows full inhibitory capability against bovine trypsin at equimolar concentration. **C.**, **D.** Reduced proliferation in UWB1.289 cells **C.** and OVCA420 cells **D.** after transduction with SPINK1-targeted shRNA (KD1) is rescued by treatment with rSPINK1 at concentrations indicated, as assessed by EdU assays. UWB1.289 NT *vs* KD1 *p* = 0.0062; KD1 *vs* 10 nM *p* = 0.0271; KD1 *vs* 100 nM *p* = 0.0017. For OVCA420, NT *vs* KD1 *p* = 0.037; KD1 *vs* 10 nM *p* = 0.013; KD1 *vs* 100 nM *p* = 0.0108. **E.**, **F.** CAOV3 and OVCAR3 cells treated with different concentrations of rSPINK1 show dose dependent increases in proliferation as assessed by EdU assays. (CAOV3: 10 nM, *p* = 0.0246, 100 nM *p* < 0.0001, OVCAR3: 10 nM *p* = 0.0183, 100 nM *p* = 0.0137.) *p < 0.05; **p < 0.01; ***p < 0.0001 (unpaired *t*-test).

SPINK1 is a soluble factor secreted by cancer cells, and we anticipate that its effects on proliferation are likely to be mediated through an autocrine signaling pathway. To evaluate whether similar effects on growth could be achieved using exogenous SPINK1, we expressed rSPINK1 in HEK293E cells and purified the recombinant protein to homogeneity from conditioned medium (Figure 2A). As a measure of the correct folding and biological activity of the recombinant protein, we titrated rSPINK1 based on its ability to inhibit bovine trypsin (Figure 2B).

Using the EdU proliferation assay, we determined for both UWB1.289 and OVCA420 cells that the reduction in proliferation resulting from shRNA knockdown of endogenous SPINK1 could be reconstituted in a dose-dependent fashion by treatment with rSPINK1 (Figure 2C, 2D). The concentrations of rSPINK1 required for reconstitution are higher than those measured for endogenous SPINK1 in conditioned media; this might be explained if endogenous SPINK1 is secreted near its site of action, achieving high local concentrations at the cell surface. Alternatively, it is possible that the C-terminal His-tag on rSPINK1 weakens its interaction with a critical receptor.

To determine whether the capacity of SPINK1 to drive proliferation is unique to ovarian cancer cell lines in which SPINK1 is normally expressed or is a more general property, we next assessed potential proliferation differences induced by rSPINK1 in cell lines that had very low endogenous SPINK1 expression levels. When CAOV3 and OVCAR3 cells were treated with different concentrations of rSPINK1 (10 nM and 100 nM), we found significant concentration dependent increases in proliferation for both cell lines (Figure 2E, 2F). These results suggest that the signaling pathway(s) through which SPINK1 drives proliferation are generally active and functional in ovarian cancer cells and can potentially be influenced by exogenous SPINK1 protein levels.

### SPINK1 effects on proliferation are mediated through EGFR signaling

**Figure 3:**
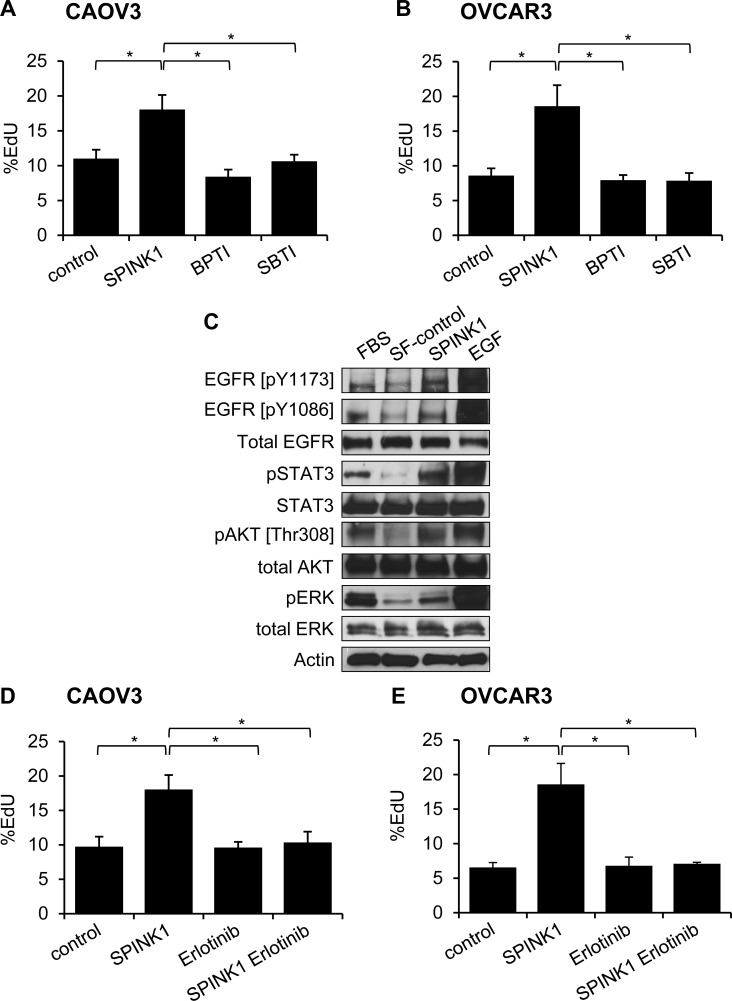
Molecular mechanisms of SPINK1-induced proliferation in ovarian cancer cells **A.**, **B.** Enhanced proliferation in CAOV3 cells **A.** or OVCAR3 cells **B.** treated with 100 nM SPINK1 is not recapitulated in cells treated instead with 100 nM BPTI or 100 nM SBTI, as assessed by EdU assays. CAOV3: control (untreated cells) *versus* rSPINK1 *p* = 0.0338, control *versus* BPTI NS, control *versus* SBTI NS, SPINK1 *versus* BPTI *p* = 0.018, SPINK1 *versus* SBTI *p* = 0.0297; OVCAR3: control *versus* SPINK1 *p* = 0.039, control *versus* BPTI NS, control *versus* SBTI NS, SPINK1 *versus* BPTI *p* = 0.031, SPINK1 *versus* SBTI *p* = 0.035. **C.** Western blot analysis evaluating phosphorylation of EGFR, STAT3, AKT and ERK in response to rSPINK1 treatment of OVCAR3 cells under serum-free (SF) conditions. Enhanced phosphorylation is seen in rSPINK1-treated sample relative to SF control. **D.**, **E.** Enhanced proliferation in CAOV3 cells **D.** or OVCAR3 cells **E.** treated with 100 nM rSPINK1 is blocked by simultaneous treatment of cells with 1 μM EGFR inhibitor erlotinib, as assessed by EdU assays. CAOV3 cells: control *versus* SPINK1 *p* = 0.021, control *versus* erlotinib NS, control *versus* SPINK1 erlotinib NS, SPINK1 *versus* erlotinib *p* = 0.021, SPINK1 *versus* SPINK1 erlotinib *p* = 0.0284; OVCAR3: control *versus* SPINK1 *p* = 0.025, control *versus* erlotinib NS, control *versus* SPINK1 erlotinib NS, SPINK1 *versus* erlotinib *p* = 0.0224, SPINK1 *versus* SPINK1 erlotinib *p* = 0.0314. *p < 0.05; **p < 0.01; ***p < 0.0001 (unpaired t-test with Welch's correction) NS not significant.

As SPINK1 is a known trypsin inhibitor, we evaluated whether the proliferation enhancing effects seen above could be triggered through inhibition of trypsin or a trypsin-like protease, by comparing the activity of SPINK1 with other trypsin inhibitors. Measuring proliferation in CAOV3 and OVCAR3 cells treated with rSPINK1 or with bovine pancreatic trypsin inhibitor (BPTI) or soybean trypsin inhibitor (SBTI), two alternative protein protease inhibitors of trypsin that function by mechanisms similar to SPINK1 [17], we found that the proliferative effect is induced uniquely by rSPINK1 treatment (Figure 3A, 3B). These results highlight the specificity of the proliferative effect of SPINK1 on ovarian cancer cells, and further suggest that SPINK1 triggers proliferation through a mechanism distinct from its trypsin inhibitory activity.

SPINK1 has been reported previously to stimulate proliferation through activation of EGFR signaling as a putative novel EGFR ligand in several other tumor types [10, 11, 13], but this phenomenon has not been examined in ovarian cancer models. To address this hypothesis, we treated serum starved OVCAR3 cells with rSPINK1 or epidermal growth factor (EGF) as a positive control, and evaluated cell lysates for phosphorylation of EGFR. We found that SPINK1 modestly enhanced phosphorylation of two specific autophosphorylation sites of EGFR, pY1173 and pY1086, when comparing rSPINK1 treated cultures to serum starved control cultures (Figure 3C). In addition, we detected increased phosphorylation of STAT3, AKT, and ERK (Figure 3C), important downstream effectors of EGFR signaling. As these EGFR signaling pathways have been extensively linked to proliferation in ovarian cancer [18-20], activation of these pathways by SPINK1 offers a plausible mechanism by which SPINK1 may exert its proliferative effects in cell culture.

To assess whether stimulation of ovarian cancer cell proliferation by SPINK1 is dependent on EGFR signaling, we next evaluated proliferation of CAOV3 and OVCAR3 cells treated with rSPINK1 in the presence or absence of erlotinib, a small molecule drug that selectively targets the ATP binding site of the EGFR kinase domain [21]. For both cell lines, the proliferative response of the cells to rSPINK1 was entirely blocked by the addition of erlotinib (Figure 3D, 3E). In sum, these results suggest that the major mechanism by which SPINK1 impacts cell proliferation involves EGFR signaling.

### SPINK1 mediates resistance to anoikis in ovarian cancer cells

**Figure 4:**
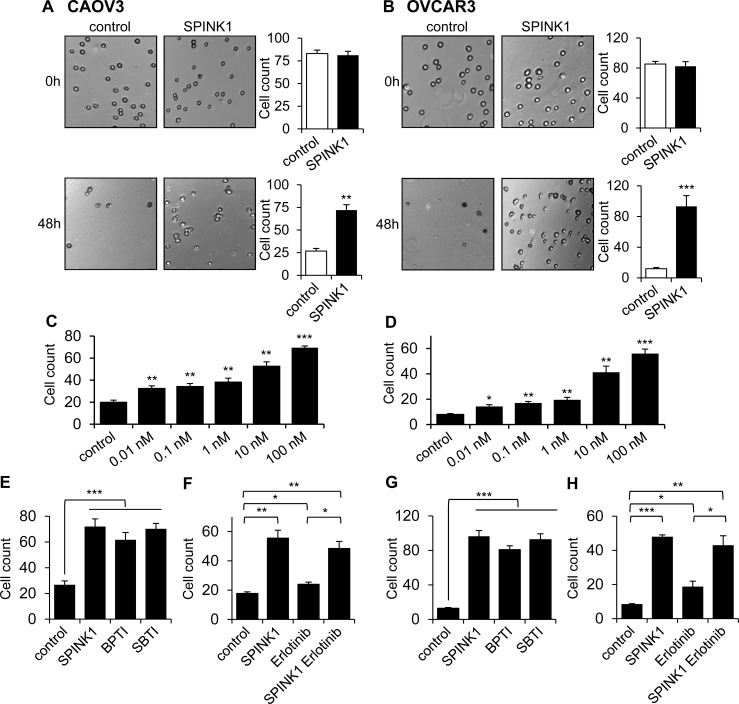
SPINK1 promotes resistance to anoikis in ovarian cancer cells **A.**, **B.** CAOV3 cells **A.** and OVCAR3 cells **B.** grown under serum free conditions on ultra-low attachment plates show reduced cell count after 48 hours in the absence of rSPINK1, while higher cell density is maintained in the presence of 100 nM rSPINK1. CAOV3: *p* = 0.001; OVCAR3: *p* = 0.0009. **C.**, **D.** CAOV3 cells **C.** and OVCAR3 cells **D.** show dose-dependent anoikis resistance after 48 h in response to increasing concentrations of rSPINK1. CAOV3: control *vs* SPINK1 0.01 nM *p* = 0.007, control *vs* 0.1 nM *p* = 0.0066, control *vs* 1 nM *p* = 0.0085, control *vs* 10 nM *p* = 0.0013, control vs 100 nM *p* < 0.0001; OVCAR3: Control *vs* SPINK1 0.01 nM *p* = 0.0171, control *vs* 0.1 nM *p* = 0.0016, control *vs* 1 nM *p* = 0.0059, control *vs* 10 nM *p* = 0.0055, control *vs* 100 nM *p* = 0.0006. **E.**, **G.** CAOV3 cells **E.** or OVCAR3 cells **G.** treated with 100 nM rSPINK1 or with 100 nM of alternative trypsin inhibitors BPTI or SBTI for 48 h show a similar degree of protection from anoikis relative to untreated control cells. CAOV3: control *versus* SPINK1, BPTI, SBTI, *p* < 0.0001 (ANOVA); OVCAR3: control *versus* SPINK1, BPTI, SBTI *p* < 0.0001 (ANOVA). **F.**, **H.** Protection from anoikis seen in CAOV3 cells **F.** or OVCAR3 cells **H.** treated with 100 nM rSPINK1 for 48 h is not significantly abrogated by simultaneous treatment with 1 μM EGFR inhibitor erlotinib. CAOV3: control *versus* SPINK1 *p* < 0.005, control *versus* erlotinib *p* = 0.016, control *versus* SPINK1 and erlotinib *p* = 0.0061, erlotinib *versus* SPINK1 plus erlotinib *p* = 0.0108; OVCAR3: control *versus* SPINK1 *p* < 0.0001, control *versus* erlotinib *p* = 0.0475, control *versus* SPINK1 and erlotinib *p* = 0.0082, and erlotinib *versus* SPINK1 plus erlotinib *p* = 0.0136. *p < 0.05; **p < 0.01; ***p < 0.0001 (unpaired *t*-test with Welch's correction unless otherwise specified).

Ovarian cancer metastasizes through the detachment of cells from the primary tumor and subsequent establishment of metastatic lesions on the peritoneum and omentum. This process requires ovarian cancer cells to become resistant to anoikis (apoptosis normally triggered by loss of cell-matrix interactions) in order to survive as individual cells or small clusters in the ascites fluid of the peritoneal cavity [22, 23]. To assess whether, in addition to its effect on proliferation, SPINK1 may promote cell survival under anchorage independent conditions, we measured ovarian cancer cell survival on ultra-low attachment plates in the absence and presence of rSPINK1. CAOV3 or OVCAR3 cells seeded into ultra-low attachment plates in the absence of rSPINK1 underwent substantial anoikis as compared to cultures treated with 100 nM rSPINK1 (Figure 4A, 4B). In assessing the effect on ultra-low attachment cultures of a broad range of rSPINK1 concentrations, we observed a dose-dependent protective effect, with significant protection from anoikis relative to untreated cultures at rSPINK1 concentrations as low as 10 pM (Figure 4C, 4D).

To probe the mechanism through which SPINK1 may protect ovarian cancer cells from anoikis, we assessed the ability of alternative trypsin inhibitors BPTI and SBTI to recapitulate the effect of rSPINK1. Intriguingly, by contrast with our assays of proliferation (Figure 3A, 3B), we found that BPTI and SBTI were both capable of protecting ovarian cancer cells from anoikis with similar potency to rSPINK1 (Figure 4E, 4G). We next evaluated the dependence of SPINK1 protection from anoikis on EGFR signaling by performing parallel anoikis assays in the presence or absence of erlotinib. For both CAOV3 and OVCAR3 cells, we found that erlotinib treatment did not significantly attenuate the anoikis protection conferred by rSPINK1 (Figure 4F, 4H). In aggregate, these results implicate a completely distinct mechanism of action for SPINK1 in protection of ovarian cancer cells from anoikis; our data demonstrate that anoikis protection is conferred via inhibition of trypsin or a trypsin-like serine protease, and does not require modulation of EGFR signaling.

### SPINK1 expression in ovarian cancer is positively associated with nonserous histology, early stage, and low grade

Having implicated SPINK1 as an autocrine factor capable of promoting ovarian cancer cell proliferation and survival, we next aimed to define the patient group in which SPINK1 may play a prominent role in driving ovarian tumor growth. We evaluated SPINK1 protein expression levels in tumors from a large patient cohort (*n* = 490) using tissue microarrays (TMAs) (Table 1, patient characteristics). The mean patient age was 61 years (range 21 to 93), and the majority of the patients were diagnosed with higher stages (76.9% stage 3 and 4) and higher grades (88.7% grade 2 and 3). At the time of our analysis, median follow-up time for these patients was 44.2 months (IQR 23.6, 71.7) and 37.3% of the patients were still alive.

**Table 1:** Patient characteristics

Characteristics	Total (N=490)
**Age at Diagnosis**	
N	490
Mean (SD)	61.5 (12.6)
Median	61.0
Q1, Q3	52.0, 71.0
Range	(21.0-93.0)
**Morphology**	
Serous	342 (69.8%)
Mucinous	16 (3.3%)
Endometrioid	82 (16.7%)
Clear cell	29 (5.9%)
Mixed Epithelial	21 (4.3%)
**Stage**	
1	79 (16.1%)
2	34 (6.9%)
3	301 (61.4%)
4	76 (15.5%)
**Grade**	
1	25 (5.1%)
2	48 (9.8%)
3	387 (78.9%)
Unknown	30 (6.1%)
**Debulking category**	
Optimal	422 (86.1%)
Sub-optimal	54 (11.0%)
Unknown	14 (2.9%)
**Vital Status**	
Alive	183 (37.3%)
Dead	307 (62.7%)
**Diagnosis to Last Follow-up**	
N	490
Mean (SD)	50.9 (36.1) (months)
Median	44.2 (months)
Q1, Q3	23.6, 71.7
Range	(0.2-153.7)

**Figure 5:**
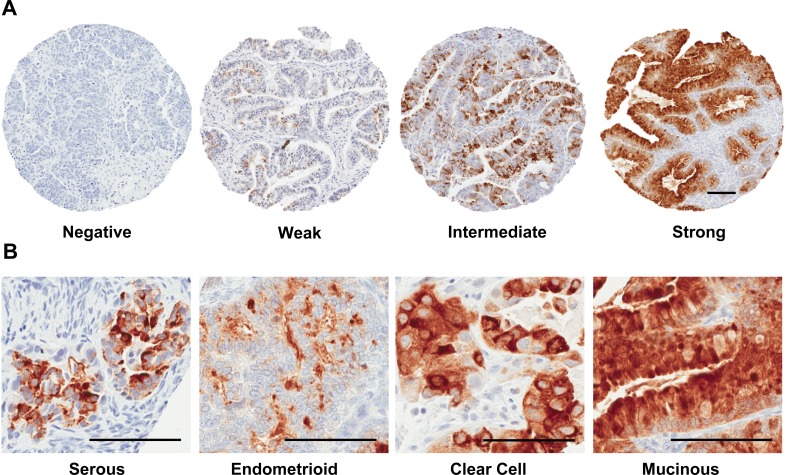
Tissue microarray staining for SPINK1 **A.** Representative tissue cores from TMAs stained for SPINK1 were scored using an automated computer algorithm with overall scores of negative, weak, intermediate or strong assigned to reflect a composite measure of intensity and extent of staining as described in the Materials and Methods section. **B.** Representative fields from positive SPINK1 staining spots derived from tumors belonging to the four histological subgroups: serous, endometrioid, clear cell, and mucinous. All Scale bars 100 μm.

SPINK1 staining varied widely in intensity and extent, and so initially SPINK1 expression was evaluated using a numerical scoring system that represented a composite measure of intensity and extent (see Materials and Methods; Figure 5A). Examples of no staining, weak staining, and strong staining were present within all morphological classifications of ovarian cancer (Figure 5B, Table 2). Incidence of SPINK1 staining positivity is considerably lower in our TMA analysis (7.8%) than the 30% reported previously in a Finnish cohort of ovarian cancer patients [16], but the trend we see of highest positivity in mucinous tumors (62.5%), followed by clear cell (13.8%), endometrioid (9.8%), mixed epithelial (9.5%) and serous tumors (4.1%), is qualitatively consistent with the previous report. Because overall positivity for SPINK1 was low, we dichotomized SPINK1 expression (present *vs*. not present) for most subsequent analyses to give greater power. When morphologies were grouped as serous *versus* nonserous tumors, we found significant positive association of SPINK1 expression with nonserous morphology (Table 3; *p* < 0.0001, Chi-square). In analysis of patients grouped by tumor stage (1/2 *versus* 3/4), SPINK1 expression in tumors was significantly associated with earlier stage (Table 3; *p* = 0.007, Chi-square). Similarly, in analysis of patients grouped by tumor grade (1 *versus* 2/3), SPINK1 expression in tumors was significantly associated with low grade (Table 3; *p* < 0.0001, Chi-square).

**Table 2:** SPINK1 expression by morphological subtype

	Negative	Weak	Intermediate	Strong	Total
Serous	328	8	2	4	342
Mucinous	6	1	5	4	16
Endometrioid	74	2	3	3	82
Clear cell	25	3	0	1	29
Mixed Epithelial	19	1	0	1	21
Total	452	15	10	13	490

**Table 3:** SPINK1 association with morphology, stage†, and grade‡

	Morphology			Stage^†^			Grade^‡^		
SPINK1 Score	Nonserous	Serous	Total	Early Stage	Advanced Stage	Total	Low Grade	High Grade	Total
**None**	124	328	452	97	355	452	16	410	426
**Positive**	24	14	38	16	22	38	9	25	34
**Total**	148	342	490	113	377	490	25	435	460
Chi square	p<0.0001			p=0.0001			p<0.0001		

† Early stage = 1,2; advanced stage = 3,4

‡ Low grade = 1; high grade = 2,3

Given that SPINK1 promoted ovarian cancer cell growth and survival in our cell culture models, SPINK1 staining might have been expected to correlate with later stage and higher grade, rather than the associations with earlier stage and lower grade that we observed. This discrepancy might be explained if the associations with stage and grade are an indirect consequence of the higher incidence of SPINK1 staining in histological subtypes more often diagnosed at early stage and low grade. In further analysis, we found no association of SPINK1 with stage or grade within the histologically-defined subset of serous tumors, and likewise found no association with stage or grade when analyzing the subset of nonserous tumors (not shown). These results suggest that the association of SPINK1 with stage and grade in the larger cohort is driven by association with nonserous morphology.

### SPINK1 expression is an independent prognostic factor for poor survival in ovarian cancer patients

**Figure 6:**
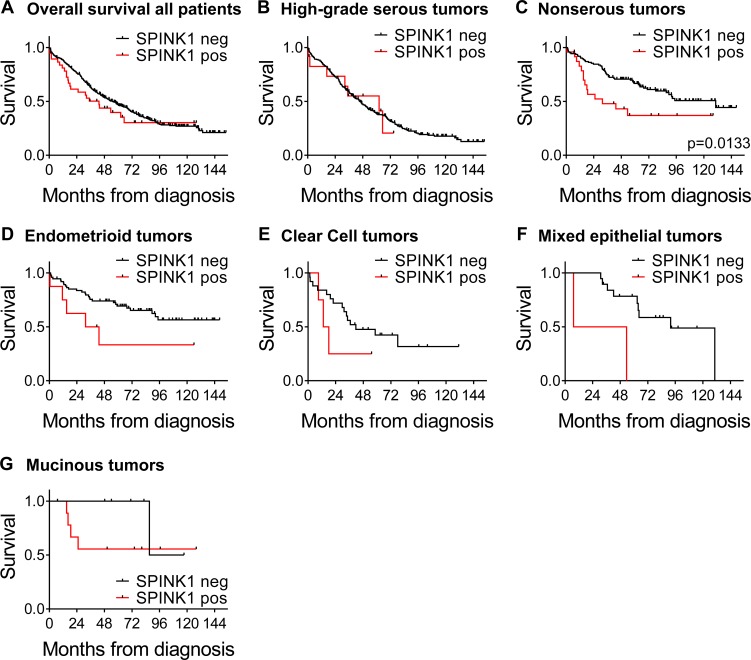
Kaplan-Meier ovarian cancer survival curves by SPINK1 positivity for all patients and morphological subgroups **A.** Overall survival for all patients comparing SPINK1-positive (red) *versus* SPINK1-negative (black), *n* = 490. **B.** Survival by SPINK1 positivity in patient subset of high-grade serous tumors, *n* = 320. **C.** Survival by SPINK1 positivity in patient subset of nonserous tumors, *p* = 0.0133, *n* = 148, **D.** Survival by SPINK1 positivity in patient subset of endometrioid tumors, *n* = 82. **E.** Survival by SPINK1 positivity in patient subset of clear cell tumors, *n* = 29. **F.** Survival by SPINK1 positivity in patient subset of mixed epithelial tumors, *n* = 21. **G.** Survival by SPINK1 positivity in patient subset of mucinous tumors, *n* = 16..

We next examined SPINK1 in terms of overall survival looking at SPINK1-positive (*n* = 38) *versus* SPINK1-negative (*n* = 452). Although there appeared to be a trend of poorer survival at earlier time points for patients with SPINK1-positive tumors, the difference did not reach significance in the overall analysis (HR 1.30, *p* = 0.2324; Figure 6A; Table 4). However, adjusting for morphology, stage, and optimal debulking revealed a significant association of SPINK1 with poor survival (HR of 1.90, *p* = 0.0045; Table 4), identifying SPINK1 as an independent prognostic factor.

**Table 4:** Cox proportional hazard model for full cohort unadjusted and adjusted for clinical variables

Variables	HR (95% CI)	*p*-value
**Unadjusted**		
SPINK1: Positive	1.30 (0.85 – 1.98)	0.2324
**Adjusted for Morphology, Stage, and Debulking**		
SPINK1: Positive	1.90 (1.22 – 2.96)	0.0045
Morphology: Serous	1.20 (0.88 – 1.65)	0.2521
Stage: Advanced	3.34 (2.23 – 5.00)	<0.0001
Debulking: Optimal	0.52 (0.38 – 0.72)	<0.0001

To investigate whether the association of SPINK1 with poor patient survival is driven by strong association within one or more specific tumor subtypes, we next analyzed association of SPINK1 staining with overall survival in patient subsets defined by tumor morphology. Among patients with high-grade serous ovarian cancers, similar survival was observed between groups with SPINK1-positive and SPINK1-negative tumors (Figure 6B). By contrast, among patients with nonserous epithelial ovarian cancers, those with SPINK1-positive tumors showed significantly poorer survival than those with SPINK1-negative tumors (Figure 6C; *p* = 0.0113). Similar to our results with the full cohort, we found that adjusting for stage and optimal debulking in nonserous subjects revealed yet stronger association between tumor SPINK1 expression and patient survival (HR 2.80, *p* = 0.0010; Table 5). This identifies SPINK1 as an independent prognostic factor in nonserous ovarian cancers, and suggests that SPINK1 expression may precede tumor progression and identify a subset of early stage cancers with poor prognosis.

**Table 5:** Cox proportional hazard model for nonserous tumors unadjusted and adjusted for clinical variables

Variables	HR (95% CI)	*p*-value
**Unadjusted**		
SPINK1: Positive	2.12 (1.17 – 3.85)	0.0133
**Adjusted for Stage, and Debulking**		
SPINK1: Positive	2.80 (1.51 – 5.19)	0.0010
Stage: Advanced	4.55 (2.59 – 7.99)	<0.0001
Debulking: Optimal	0.22 (0.07 – 0.66)	0.0067

Upon further subdivision of nonserous patients into individual morphologies in an exploratory fashion, although the numbers of SPINK1-positive tumors and events in the subdivided groups are quite low, trends of poorer survival among SPINK1-positive patients are reflected in all nonserous morphological subgroups (endometrioid, clear cell, mixed epithelial, and mucinous, Figure 6D-6G, respectively). We conclude from these analyses that SPINK1 is prognostic for poor survival specifically in nonserous subtypes of epithelial ovarian cancer, and within this broad category, the association of SPINK1 with poor survival spans multiple morphological subtypes.

## DISCUSSION

In the present study, we have identified SPINK1 as a tumor-produced autocrine factor that can contribute to both the proliferative potential and the anoikis resistance of epithelial ovarian cancer cells, and thus plays a functional role in driving ovarian cancer growth and progression. We have also evaluated the expression of tumor SPINK1 protein in a large cohort of patients with ovarian cancer and have assessed associations with tumor morphology, stage, grade, and overall survival. Consistent with our functional studies in cell culture models, our clinical data reveal that SPINK1 tumor staining is an independent prognosticator of poor patient survival, and further, that SPINK1 expression identifies a subset of nonserous epithelial ovarian cancers with particularly poor prognosis.

Within the past decade, morphological subtypes of epithelial ovarian cancer have come to be appreciated as distinct diseases, arising from different cells of origin and driven by different initiating mutations. For example, high-grade serous ovarian cancers are believed to derive from the fimbrial epithelium [24, 25], and most often feature loss of *TP53*, as well as mutations of *BRCA1* or *BRCA2* [26, 27]. Endometrioid and clear cell ovarian tumors are associated with endometriosis, which is believed to represent a precursor lesion of these cancer subtypes on the basis of similar mutational and molecular expression profiles [26, 28]. By contrast, mucinous ovarian tumors have expression profiles closer to those found in colonic epithelium [29]. The most intense focus of recent research has been on high-grade serous ovarian cancers, which comprise the largest category of invasive epithelial ovarian tumors (∼70%) and as a group tend to be diagnosed at later stage with poor outcomes [25]. Comparatively understudied, nonserous tumor subtypes are more often diagnosed at an earlier stage when they can be successfully surgically removed, but if the cancers progress, they still account for a significant proportion of ovarian cancer deaths [26, 30, 31], as is evident in our cohort (Figure 6). Current treatments do not target specific molecular drivers of these less common tumor subtypes, nor are prognostic biomarkers in clinical use tailored toward the specific tumor subtypes [30, 31].

In the present study, while our cohort was comprised of patients with a typical distribution of ovarian tumor subtypes, we found SPINK1 to be associated with poor survival specifically in the subset of patients with nonserous ovarian cancers. Our results suggest that SPINK1 could have clinical utility as a prognostic tissue biomarker in patients with these relatively rare subtypes of ovarian cancer. In patient studies on breast and prostate cancers [12, 32], high levels of SPINK1 staining in tumor tissue have been similarly associated with poor prognosis in specific patient subsets. In breast cancer, SPINK1 staining was reported to be widespread in breast tumors, with high SPINK1 associated with poor distant metastasis free survival specifically in estrogen receptor positive (ER+) breast cancer patients [14]. SPINK1 staining is present with lower incidence (approximately 10%) in prostate cancer, where it was reported as a marker for a particularly aggressive molecular subtype associated with poor progression free and recurrence free survival [32, 33], although subsequent studies using different cohorts have not uniformly confirmed the association with clinical outcome [34-36]. For ovarian cancer, use of SPINK1 as a biomarker may be best accomplished through staining of larger tumor sections, as one limitation of our study was the use of TMAs to detect SPINK1 protein expression, where the heterogeneous expression of SPINK1 in ovarian tumors may have led to false negatives (SPINK1 expression missed due to inadequate tumor sampling). This could help to explain the lower incidence of SPINK1 positivity in our cohort compared with an earlier study [16]. As an alternative approach, it may be useful to assess SPINK1 in serum or urine, which have shown potential utility for diagnosis, preoperative prognostication, and post-treatment surveillance [37-39].

Beyond its potential use as a prognostic marker, our findings implicating SPINK1 as a functional driver of ovarian cancer cell growth and survival suggest that it may offer a target for pharmacological intervention in the subset of patients with SPINK1-expressing tumors. In our functional studies, we found that even very low-expressing ovarian cancer cells can show strong dependence of proliferation on SPINK1. Likewise, cancer cells were significantly protected from anoikis at SPINK1 concentrations as low as 10 pM. These observations suggest that SPINK1 may significantly drive ovarian cancer growth and progression even for tumors with modest expression levels. This idea is also consistent with our patient biospecimens analysis, in which we found that SPINK1 positivity, irrespective of staining intensity, was significantly associated with poorer survival.

Intriguingly, our data point to dual mechanisms by which SPINK1 can promote ovarian cancer growth and progression: it appears to stimulate proliferation through an EGFR-dependent mechanism, while promoting resistance to anoikis through a serine protease-dependent, EGFR-independent mechanism. EGFR is known to be highly expressed in the majority of ovarian cancers, but phase II/III trials of EGFR inhibitors have not shown favorable clinical outcomes among unselected cohorts of ovarian cancer patients, or among any subgroups thus far identified [18, 40]. It is possible that SPINK1 and EGFR coexpression might help to identify a subset of patients more likely to benefit from existing EGFR-targeted therapies (e.g. cetuximab, erlotinib, panitumumab), however these agents would not block the pathway through which SPINK1 confers protection from anoikis. Alternatively, it may be that a more complete blockade of the oncogenic activities of SPINK1 in ovarian cancer can be achieved through direct targeting of SPINK1 itself. Of note, preclinical studies in a mouse model of SPINK1-driven prostate cancer showed therapeutic benefit from a function-blocking SPINK1 antibody [10].

Efforts to therapeutically target the oncogenic activities of SPINK1 would be aided by a better understanding of the molecular interactions involved in these activities. Previous studies have implicated a direct physical interaction between SPINK1 and EGFR; endogenous EGFR from cancer cell lysates was shown to coimmunoprecipitate with exogenous rSPINK1 [10, 13], and direct binding of EGFR extracellular domain to rSPINK1 was also demonstrated using a quartz-crystal microbalance technique [13]. Despite the evidence for this interaction, information is lacking about the structural nature of the protein complex and the specific epitopes involved. Early reports based on incomplete amino acid sequences mistakenly speculated the sequence and structural similarity and evolutionary relatedness of EGF and SPINK1 [41, 42], leading to confusion that continues to propagate through the modern literature. However, X-ray crystallographic analyses [43-45] have since revealed these small proteins to possess completely dissimilar three-dimensional structures. Because the surface epitopes of SPINK1 do not resemble those of EGF or other EGFR ligands, it cannot be assumed that it interacts with EGFR in the same manner or at the same site as other ligands. It is also not known whether the SPINK1 epitope responsible for interaction with EGFR encompasses or overlaps with the canonical binding loop that is involved in serine protease inhibition. Thus, design of rational therapeutic interventions to optimally target the interaction of SPINK1 with EGFR would benefit from efforts to better define the molecular details of the interaction.

Our discovery that SPINK1 protects ovarian cancer cells from anoikis, and that other similar trypsin inhibitors can recapitulate this effect, likewise poses additional questions, chiefly among them the identity of the protease target(s) of SPINK1 responsible for this effect. The natural physiological targets of SPINK1 in the pancreas are trypsin 1 (cationic trypsin) and trypsin 2 (anionic trypsin) [5]; both of these enzymes can be expressed by ovarian tumors, and in this context are known as tumor-associated trypsin 1 and 2, respectively [46]. It is possible that trypsin inhibition by SPINK1 results in resistance to anoikis, however this would be counterintuitive, because the established roles for tumor-associated trypsins that have been documented to date are largely of a pro-tumorigenic nature [46], and tumor-associated trypsin secretion has been linked with more malignant ovarian cancers and poorer patient outcomes [16, 47]. Trypsins represent only a few of the potential targets of SPINK1, as the human proteome contains about 80 active serine proteases in the S01 family with tryptic-like specificity for cleavage after Lys or Arg residues (Merops Peptidase Database [48]), any of which may represent potential targets for inhibition by SPINK1. Intriguingly, anoikis resistance in ovarian cancer has been previously linked with downregulation of another serine protease, HtrA1 [49]; however, this enzyme appears unlikely to be a direct target of SPINK1 since its primary substrate specificity directs cleavage following small hydrophobic or polar residues [50], but not after Lys or Arg as is typical of protease targets of SPINK1. It is possible that HtrA1 and the direct target of SPINK1 signal through a common pathway and may be linked through common regulatory interactions, as often seen in proteolytic networks that comprise the “protease web” [51]. Further studies to unravel the nature of the protease-dependent anoikis protection conferred by SPINK1 may aid the ultimate development of therapeutic interventions targeting this activity.

The key advances of the present study are the identification of SPINK1 as a driver of proliferation in ovarian cancer cell lines as well as a factor responsible for protecting ovarian cancer cells from anoikis. Given that ovarian cancer progresses through dissemination, establishment and growth of prolific metastases on the peritoneum and omentum, these novel functions of SPINK1 assist in multiple stages of progression, via multiple mechanisms that here we have begun to elucidate. In addition, our work suggests that SPINK1 may have clinical relevance as a biomarker particularly in currently understudied nonserous ovarian cancer subtypes. Importantly, our studies not only provide insight into mechanisms of tumor progression, but also suggest new avenues for development of molecularly targeted therapies.

## MATERIALS AND METHODS

### Cell culture

CAOV3, OVCAR3, and UWB1.289 cell lines were purchased from ATCC (Manassas, VA). OVCA420 was a gift to JAC from Dr. Robert C. Bast Jr. (University of Texas MD Anderson Cancer Center). OVCA420 and CAOV3 cells were maintained in high glucose Dulbecco's modified eagle medium (DMEM; Gibco, Invitrogen, Carlsbad, CA) supplemented with sodium pyruvate (0.5mM, Gibco, Invitrogen), 5 % fetal bovine serum (FBS; Biosera, Kansas City, MO), 1X NEAA (MEM Non-Essential Amino Acids, Gibco, Invitrogen) and 1% PSA (Penicillin-Streptomycin-Amphotericin B, MP Biomedicals, Santa Ana, CA). UWB1.289 and OVCAR3 cells were grown in Mammary Epithelial Cell Growth Medium (MEGM bullet kit, Lonza, Walkersville, MD) mixed 1:1 with DMEM supplemented as described above. All cell lines were grown at 37°C in a humidified atmosphere with 5% CO_2_.

### Lentiviral transduction

Lentiviral short hairpin RNA (shRNA) constructs NM_003122.2-158s1c1 (KD1) and NM_003122.2-260s1c1 (KD2) targeting human SPINK1 were obtained from the MISSION TRC-Hs 1.0 and 1.5 libraries (Sigma, St. Louis, MO). Three additional constructs were initially tested and determined to possess less efficient knock-down capability (data not shown). A non-target control shRNA recognizing no human genes was used as a negative control in all RNAi experiments. Infective lentivirus particles were produced using HEK 293FT cells following supplier protocols. For transduction of OVCA420 and UWV1.289, 10^6^ cells were seeded in 10 cm^2^ culture dishes. After 24 h, the medium was replaced with 3.6 ml fresh medium mixed with 2.4 ml of lentiviral particle-containing conditioned medium and 3.6 μg/ml polybrene (EMD MILLIPORE Merck KGaA, Darmstadt, Germany). The medium was changed again after 24 h and transduced cells were selected with 1 μg/ml puromycin (Corning, Kennebunk, ME).

### RNA extraction and quantitative real-time PCR

RNA was isolated using TRIzol reagent (Invitrogen) according to the manufacturer's protocol. RNA concentration was determined using the Nanodrop ND-1000 spectrometer at an absorbance of 260/280. The High Capacity cDNA Reverse Transcription Kit (Applied Biosystems, Foster City, CA) was used to synthesize cDNA. Quantitative real-time PCR was completed on reverse-transcribed cDNA using the ABI 7900HT Fast-Real Time PCR System. Taqman assays for SPINK1 (Hs00162154_m1) and GAPDH (Hs99999905_m1) were purchased from Applied Biosystems and were run over 40 cycles. Data were analyzed using SDS RQ Manager Software (Applied Biosystems).

### Enzyme-linked immunosorbent assay (ELISA)

Conditioned media from all four cell lines (OVCA420, UWB1.289, CAOV3, OVCAR3) were tested using the human SPINK1/TATI PicoKine ELISA kit (#EK1241, Boster Biological, Pleasanton, CA). Briefly, cells were grown to near confluency, serum-containing media were replaced with serum-free media, media were collected after 48 h incubation and concentrated 10-fold using Centricon filtration devices (3000 MWCO, EMD Millipore). Media were diluted 2-fold with assay buffer and dispensed in quadruplicate alongside manufacturer-provided standards in 96-well plates precoated with SPINK1 monoclonal antibody. Washes and subsequent incubations with biotinylated detection antibody, avidin-biotin-peroxidase complex, 3,3′,5,5′-tetramethylbenzidine color developing agent and stop solution were performed according to the kit manufacturers' instructions, and absorbance was read at 450 nm in a SpectraMax M5 plate reader (Molecular Devices). Standard curve and calculation of SPINK1 concentrations in conditioned media were performed with Prism 6.0.

### Recombinant SPINK1 expression, purification, and characterization

Recombinant human SPINK1 protein (rSPINK1) was expressed in HEK293E cells transiently transfected with a SPINK1 minigene construct in pcDNA3.1(−) possessing intron 1 and a C-terminal 10×His-tag [52]. Cells were transfected by a linear polyethylenimine (PEI) transfection reagent using a protocol previously developed for the human tissue inhibitor of metalloproteinases 1 [53, 54]. Briefly, cells were seeded the day prior to transfection at 2×10^7^ per 150 cm^2^ T-flask in DMEM containing 10% FBS and 50 μg/ml G418 (Corning). Transfection reagent was prepared for each flask by mixing 200 μg PEI into 100 μg DNA in 4 ml 150 mM NaCl, incubating at room temperature for 30 min, then mixing with 25 mL of DMEM containing 2.5% FBS. Cells were treated with the transfection mixture for 24 h and then medium was replaced with serum free DMEM. Conditioned medium was collected after 5 days and His-tagged protein was recovered by binding to Ni-NTA agarose beads (Qiagen) overnight. Beads were transferred into a disposable gravity flow column (BioRad, Hercules, CA), washed with 150 mM NaCl, 50 mM NaH_2_PO_4_, 10 mM imidazole pH 8.0, and eluted with 300 mM NaCl, 50 mM NaH_2_PO_4_, 250 mM imidazole pH 8.0. Affinity purified rSPINK1 was further purified to homogeneity via gel filtration chromatography (Superdex 75, GE Healthcare) in 300 mM NaCl, 100 mM Tris pH 8.0. Activity of purified rSPINK1 as a trypsin inhibitor was confirmed, and concentration of active protein assessed, by titration against bovine trypsin (Sigma) as previously described [55]. Intact mass and sequence of purified rSPINK1 were confirmed by intact mass measurements using nanoflow liquid chromatography electrospray mass spectrometry and by protein identification from tryptic digests using nanoflow liquid chromatography tandem mass spectrometry conducted at the Proteomics Core, Mayo Clinic Rochester.

### MTT viability assay

To measure cell viability, 6-9 replicates of 5000 cells per well were plated in 96 well plates, grown overnight and then incubated with 5 mg/ml 3-(4,5-dimethylthiazol-2-yl)-2,5-diphenyl tetrazolium bromide (MTT, Amresco) at 37°C for 4 h following the manufacturer's protocol. Formazan crystals were dissolved for 20 h in 10% SDS and then absorbance at 560nm was measured using a SpectraMax M5 plate reader (Molecular Devices). Graphed data show average and SEM of biological replicates after subtraction of baseline reading for media control.

### EdU proliferation assay

The fraction of cells actively undergoing DNA synthesis was evaluated using 5-ethynyl-2′-deoxyuridine (EdU) incorporation assays. OVCA420, UWB1.289, OVCAR3, or CAOV3, in some experiments with shRNA knockdown of SPINK1 as described, were seeded in 6-well plates at 1×10^5^ cells per well (OVCA420, UWB1.289, or CAOV3) or 3×10^5^ cells per well (OVCAR3). The next day media were changed to serum-free media and in some experiments cells were treated with varying concentrations of rSPINK1. After 24 hours, EdU incorporation was conducted using the Click-iT EdU Alexa Fluor 488 kit (Invitrogen). Following a 2 h interval of EdU incorporation, cells were fixed with 3.7% formaldehyde in phosphate buffered saline (PBS), permeabilized with 0.5% Triton X-100 in PBS, reacted with Alexa Fluor 488 azide, and nuclei were counterstained with Hoechst 33342, all according to manufacturer instructions. In some experiments, cells were subsequently blocked with 5% nonfat dry milk in PBS for 10 minutes at RT, incubated overnight at 4°C with 1:200 monoclonal anti β-tubulin clone TUB 2.1 (Sigma-Aldrich cat # T4026), and then stained with 1:500 goat anti mouse Alexa Flour 546 for 30 min at room temperature. Cells were rinsed with PBS and pictures were taken at 40× magnification for manual cell counting and calculation of proliferative indices.

### Western blot analysis

OVCAR3 cells (10^6^) were seeded in 10 cm plates and grown overnight. One control plate was maintained in complete media while other plates were serum starved overnight. After 16 h cells were treated with 10 nM rSPINK1 or 100 ng/ml EGF (Peprotech, Rocky Hill, NJ) for 15 min and then cells were lysed in 1% Triton-X, 150 mM NaCl, 10 mM Tris, pH 7.9, 1 mM EDTA, 1 mM EGTA. Protein concentration determinations were performed in triplicate using the bicinchoninic acid (BCA) assay (Pierce, Thermo Scientific) following the manufacturer's protocol and absorbance was measured with the SpectraMax M5 Multi Mode Microplate reader. Concentration adjusted protein samples were run on 4-20% gradient SDS gels (BioRad) and transferred to nitrocellulose membrane. Blots were probed with primary antibodies (pEGFR 1173 Cell Signaling (CS) #4407S, pEGFR 1086 Epitomics #1139S, total EGFR Abcam ab52894, pAKT Thr308 CS #9275L, AKT 1/2/3 (H-136) Santa Cruz #sc8312, pERK CS #43705, panERK CS #9102L, pSTAT3 Tyr705 CS #91455, panSTAT3 CS #9132, Actin Santa Cruz #sc 1616) in Tris buffered saline containing 0.05% Tween-20 (TBST) and 5% BSA overnight. Blots were washed in TBST and incubated with secondary antibody for 1 h (HRP goat anti rabbit Invitrogen #656120, HRP Donkey anti-goat Santa Cruz #sc 2020), washed again and visualized with Clarity Western ECL (BioRad) and exposure to film.

### Anoikis resistance assay

CAOV3 and OVCAR3 cells (8×10^4^) were plated in 6-well ultra-low attachment plates (Corning) under serum free or varying treatment conditions (rSPINK1, BPTI, SBTI, erlotinib) and imaged via microscopy (16X magnification) initially, after 24 h and 48 h. Four representative photographs from each well were taken at each time point and cells were counted and averaged.

### Study population

Tumor biospecimens used for this study were derived from a Mayo Clinic cohort of 570 patients. Eligible patients were women 20 years or older diagnosed with pathologically confirmed primary invasive epithelial ovarian cancer between 1999 and 2009 at Mayo Clinic's gynecologic surgery and medical oncology departments. All protocol procedures and patient contact materials were reviewed and approved by the Institutional Review Board of the Mayo Clinic. All study patients provided written informed consent. Data on clinical features, histology, and surgical outcome were extracted from medical records by experienced research nurses under supervision of gynecologic and medical oncologists. Further details about this cohort have been described previously [56, 57].

### Tissue microarrays and immunohistochemistry

Tissue microarrays of patients' formalin-fixed paraffin embedded (FFPE) tumor biospecimens were acquired through the Mayo Clinic Ovarian Cancer SPORE and have been previously described [56]. Briefly, five TMAs, each containing about 350 0.6 mm cores (three randomly placed cores per tumor) were constructed using an automated Beecher Instruments ATA-27 arrayer. Sections (5 μm) were cut and mounted on charged slides. Following deparaffinization and rehydration, antigen was retrieved in citrate buffer, endogenous peroxidase was blocked with 3% H_2_O_2_ and slides were incubated with serum-free protein block (Dako). Slides were then stained for 1 h at room temperature with SPINK1 monoclonal antibody (Novus #H00006690-M01) followed by 30 min with secondary anti-mouse labeled polymer/horse radish peroxidase conjugate (Dako #K4007) and then color was developed using 3,3′-diaminobenzidine (DAB, EnVision+, Dako).

Slides were scanned using a ScanScope scanner (Aperio Technologies, Vista, CA), and reviewed for tissue quality and presence of tumor by one reviewer (CM) in consultation with a gynecologic pathologist (AN). Damaged tissue and TMA spots with fewer than 30 tumor cells were excluded from analysis. Spots were scored using Image Scope Software (Aperio Technologies) implementing the positive pixel count algorithm, which assigned to each pixel a value of negative, weak, intermediate, or strong intensity. A composite score for each spot reflecting both intensity and extent of staining was assigned as follows: negative, no staining; weak, 1-10% intermediate or 1-5% strong intensity pixels; intermediate, 10-40% intermediate or 5-10% strong intensity pixels; strong, >40% intermediate or >10% strong intensity pixels. A small number of spots (10) showed very weak diffuse acellular staining with no pixels reaching intermediate or strong intensity; these were judged to represent a background artifact and were classified as negative. When different staining scores were assigned for replicate spots from a single patient, the maximum value was used for analysis. Out of 570 patients, 70 were excluded due to missing or damaged tissue, and 10, with tumor morphology classified as non-epithelial ovarian, borderline, or unknown, were excluded for histological criteria. Tumor morphologies included in the analyses were serous, endometrioid, clear cell, mucinous, and mixed epithelial.

### Statistical analysis

Statistical comparisons for the cell culture studies were conducted with Prism version 6.0 (GraphPad Software) using the t-test. Statistical analyses for the human biospecimens data were done using the R statistical software package (version 3.1.1). Associations between SPINK1 and morphology, stage, and grade were assessed via contingency tables and the Chi-square test. Association of overall survival with a positive (weak, intermediate, strong) *versus* negative SPINK1 score was assessed via Kaplan Meier curves and Cox proportional hazards models. Models were run both unadjusted and adjusted for morphology (serous *vs* nonserous), stage (early *vs* advanced), and debulking status (sub-optimal *vs* optimal).
